# Developmental potential of surplus morulas with delayed and/or incomplete compaction after freezing-thawing procedures

**DOI:** 10.1186/s12958-019-0535-2

**Published:** 2019-10-30

**Authors:** Ni-Chin Tsai, Yu-Ting Su, Yu-Ju Lin, Hsin-Ju Chiang, Fu-Jen Huang, Fu-Tsai Kung, Kuo-Chung Lan

**Affiliations:** 1 Department of Obstetrics and Gynecology, Kaohsiung Chang Gung Memorial Hospital and Chang Gung University College of Medicine, 123 Ta-Pei Road, Niao-Sung District, Kaohsiung city, Taiwan; 2 Graduate Institute of Clinical Medicine, College of Medicine, Kaohsiung Medical University, Kaohsiung, Taiwan; 3 Department of Obstetrics and Gynecology, Xiamen Chang Gung Hospital, Xiamen, Fujian China; 4 Center for Menopause and Reproductive Medicine Research, Kaohsiung Chang Gung Memorial Hospital and Chang Gung University College of Medicine, Kaohsiung, Taiwan

**Keywords:** Blastocyst formation, Fragmentation, Frozen embryo transfer, Slow developing morulas, Surplus morulas, Vitrification

## Abstract

**Background:**

Morulas with delayed growth sometimes coexist with blastocysts. There is still limited evidence regarding the optimal disposal of surplus morulas. With the advancement of vitrification, the freezing-thawing technique has been widely applied to zygotes with 2 pronuclei, as well as embryos at the cleavage and blastocyst stages. The freezing of morulas, however, has rarely been discussed. The purpose of this study was to investigate whether these poor-quality and slow-growing morulas are worthy of cryopreservation.

**Methods:**

This is a retrospective, observational, proof-of-concept study. A total of 1033 day 5/6 surplus morulas were cryopreserved from January 2015 to December 2018. The study included 167 women undergoing 180 frozen embryo transfer cycles. After the morulas underwent freezing-thawing procedures, their development was monitored for an additional day. The primary outcome was the blastocyst formation rate. Secondary outcomes were clinical pregnancy rate, live birth rate and abortion rate.

**Results:**

A total of 347 surplus morulas were thawed. All studied morulas showed delayed compaction (day 5, *n* = 329; day 6, *n* = 18) and were graded as having low (M1, *n* = 54), medium (M2, *n* = 138) or high (M3, *n* = 155) fragmentation. The post-thaw survival rate was 79.3%. After 1 day in extended culture, the blastocyst formation rate was 66.6%, and the top-quality blastocyst formation rate was 23.6%. The day 5 morulas graded as M1, M2, and M3 had blastocyst formation rates of 88.9, 74.0, and 52.8% (*p* < 0.001), respectively, and the top-quality blastocyst formation rates were 64.8, 25.2, and 9.0% (*p* < 0.001), respectively. The clinical pregnancy rate was 33.6%.

**Conclusions:**

The post-thaw blastocyst formation rate was satisfactory, with approximately one-half of heavily fragmented morulas (M3) developing into blastocysts. Most of the poor-quality morulas were worth to freeze, with the reasonable goal of obtaining pregnancy and live birth. This alternative strategy may be a feasible approach for coping with poor-quality surplus morulas in non-PGS (preimplantation genetic screening) cycles.

## Background

Current sophisticated culture systems and freezing-thawing procedures have enhanced the application of blastocyst culturing and frozen embryo transfer (FET) [1, 2]. Blastocyst culturing can be used to select embryos that have undergone genome activation [3], and it avoids the risk of transferring potentially arrested embryos or high-order embryos. Morulas with delayed growth sometimes coexist with blastocysts, and surplus morulas are often present. Slow-developing embryos are placed in a *low priority* queue for transfer selection. There is still limited evidence regarding the optimal disposal of surplus morulas; options include fresh transfer, extended culture and transfer in subsequent FET cycles, among others (ex. discarding, which would be controversial) [4, 5].

The vitrification technique has been widely applied in zygotes with 2 pronuclei, 8-cell embryos, and blastocyst embryos, but little attention has been given to morulas in previous literature, especially concerning poor-quality morulas after vitrification. Few studies to date have focused on the developmental potential or freezing-thawing outcomes of embryos derived from the morula stage [3–10]. The survival rate of human day 4 morulas after freezing and thawing has been examined in a study addressing the morphological alterations and their applications in embryo selection [10]. The freezing-thawing strategy for surplus morulas at our institution was modified after 2015. Previously, after fresh blastocyst transfer, the morulas with delayed growth were cultivated until they reached the blastocyst stage or were cryopreserved. At present, to determine their growth potential, surplus morulas were thawed 1 day before FET. For the infertile couples, cryopreserving surplus morulas at the same time that blastocysts are cryopreserved is plausible, and they did not pay extra fees for that process at our institution.

Fragmentation has been associated with poor blastocyst formation and implantation rates, as well as chromosomal abnormalities [3, 11–14]. Little is known about the outcomes resulting from the use of pervasive fragmented morulas in fresh and FET cycles. The effects of delayed compaction and fragmentation on the developmental capacity of morulas in fresh cycles have been determined [9]; the finding was consistent with our experiences [15, 16], except for the day 5 heavily fragmented morulas, which had a low blastocyst formation rate in our previous observation. To our knowledge, no study to date has reported the post-thaw BFR of morulas with either delayed compaction or heavy fragmentation.

The purpose of this study was to determine whether these poor-quality and slow-growing morulas are worthy of cryopreservation by exploring their developmental capacity after freezing-thawing procedures.

## Methods

This is a retrospective, observational, and proof-of-concept study. A total of 1033 day 5/6 surplus morulas were cryopreserved from January 2015 to December 2018. Couples with vitrified surplus morulas who had returned for at least one FET cycle were included. The study included a total of 167 women undergoing 180 FET cycles. All couples completed the standard infertility workup; couples were not excluded based on age, sperm parameters or causes of infertility.

### Patient preparation in stimulated cycles, embryo management and embryo culture

The protocols used for controlled ovarian hyperstimulation, oocyte retrieval and embryo culture have been described previously [15, 16]. Briefly, women undergoing the GnRH antagonist protocol received an additional 0.25 mg/day GnRH antagonist (Ganirelix acetate: 0.25 mg, MSD; or Cetrorelix acetate: 0.25 mg, Serono), beginning when one leading follicle reached ≥14 mm in diameter and ending on the day of human chorionic gonadotropin (hCG) injection. Gonadotropin doses were adjusted during each cycle based on individual responses, which included serum estradiol (E2) concentration and sonographic monitoring of follicular growth. Following the maturation of two additional follicles, each ≥18 mm in diameter, recombinant hCG (Ovidrel; Merck, Serono, Modugno, Italy) and/or GnRH-agonist (Decapeptyl, Ferring GmbH) was administered. Oocytes were retrieved 36–38 h later by transvaginal aspiration under ultrasound guidance.

Standard IVF/ICSI procedures were used for oocyte fertilization. Fertilization was confirmed 16 to 18 h subsequent to IVF or ICSI. The embryos were evaluated on days 1, 2, 3 and 5. Embryos were cultured in G1™ medium (Vitrolife Sweden AB, Vastra Frolunda, Sweden) on days 1–3 and in G2™ medium (Vitrolife Sweden AB) on days 3–5 or 6. The incubator (Thermo Scientific HERACELL 150i) maintained the O_2_ level at 5% and the culture medium pH at 7.27 ± 0.07 [17], and the CO_2_ was at approximately 6.3% per the recommendation of the media provider (Vitrolife Sweden).

Extending embryo culture to the blastocyst stage was performed if there were three or more good quality 8-cell embryos on day 3. The top-quality embryos were selected by experienced embryologists and then were given priority for fresh cycle transfer.

The morula vitrification method was identical to the method used for blastocyst vitrification [18–20]. Because all studied morulas had delayed compaction, vitrification was performed on day 5 or 6. A single team of embryologists coordinated all the procedures, thereby ensuring that the culture protocols and the embryo freezing-thawing procedures were consistent and standardized.

### Embryo grading

Morulas were defined as embryos containing ≥16 blastomeres that were over 50% compacted. Morulas/compact stage embryos were graded using a modification of the simplified SART (Society for Assisted Reproductive Technology) embryo scoring system [21]. Each morula was scored according to its degree of compaction and fragmentation. Morula 1 (M1) was defined as a top-quality morula, with > 90% of its cell mass compacted and < 10% fragmentation. M2 has 70–90% compaction and 10–30% fragmentation, and M3 has 50–70% compaction and > 30% fragmentation.

Blastocysts were graded according to the Gardner & Schoolcraft system [22], which is based on the degree of blastocyst expansion and the morphological appearances of the inner cell mass and the trophectoderm cells. Top-quality blastocysts were defined as 3AA, 4AA, 5AA or 6AA.

A consensus of grades for the embryos was reached by two experienced embryologists who used the same criteria. If discordance occurred, there would be a meeting to produce a final decision.

### Endometrial preparation for frozen embryo transfer and embryo thawing

The endometrium was prepared either by artificial hormone replacement or by natural cycling. The embryos were transferred on day 6 of progesterone administration.

Morulas were warmed using a two-step dilution with sucrose [18] and then returned to G2™ medium for further culture until transfer. Laser-assisted hatching was performed 5 min after thawing [23, 24]. Assisted hatching with the diode laser system (FERTILASE; MTM Medical Technologies, Montreux, Switzerland) and short irradiation exposure times (4 m-second) were applied. A split of approximately one-quarter to one-third of the circumference of the zona pellucida was made. The morphological evolution and development of the embryos were then recorded. Morulas that failed to show changes consistent with recovery and those that became atrophic were discarded.

### Outcome analysis

An embryo was determined to have progressed to the blastocyst stage if blastulation had occurred and the inner cell mass was visible. The BFR and the top-quality BFR were analyzed.

All women were followed regularly until pregnancy outcomes were confirmed. Pregnancy was confirmed by detecting β-hCG in the urine 2 weeks after transfer. Biochemical pregnancy was defined as the absence of an identifiable pregnancy on ultrasound examination in combination with a positive urine or serum β-hCG test [25]. Clinical pregnancy was defined as the identification of at least one gestational sac via ultrasound at 6–7 weeks of gestation [25]. Ongoing pregnancy was defined as a viable pregnancy beyond 12 gestational weeks. Abortion was defined as a pregnancy that was terminated spontaneously before 12 gestational weeks. Live birth was defined as the delivery of a viable fetus beyond 23 gestational weeks. The implantation rate was defined as the number of gestational sacs divided by the number of embryos transferred.

### Statistical analysis

Continuous data are given as the mean ± standard deviation (SD). Student’s t-test was used to compare continuous data. Categorical variables, reported as proportions, were compared using the chi-square or Fisher’s exact test, as appropriate. Logistic regression analysis was performed for multivariate analysis. All tests of significance were two-tailed, with *p* < 0.05 defined as being statistically significant. All statistical analyses were performed with SPSS for Windows version 18.

## Results

### Embryo post-thaw survival conditions and blastocyst formation rates

During the 4-year study period, a total of 1033 day 5/6 surplus morulas (M1, *n* = 32, 12.8%; M2, *n* = 426, 41.2%; M3, *n* = 475, 46%) were cryopreserved. Of these, 347 (33.6%) surplus morulas (day 5, *n* = 329; day 6, *n* = 18) were thawed. A total of 167 women who were undergoing 180 FET cycles from January 2015 to December 2018 were included. The mean age of the patients was 35.8 ± 3.8 years. Of the thawed morulas, 84.5% were initially graded as being of poor quality (over 10% fragmentation) at the fresh cycle. The post-thaw survival rate of morulas was 79.3%. After 1 day in extended culture, the BFR was 66.6%, and the top-quality BFR was 23.6% (Table 1). The post-thaw morula atrophy rate (i.e., morulas unavailable for transfer) was 20.7% (72/347). Of these 72 atrophic morulas, 17 (23.6%) were graded as M2, and 54 (75%) were graded as M3. The cycle cancellation rate, during which no thawed embryos could be transferred, was 5.0% (9/180).

**Table 1:** Characteristics and outcomes of surplus morulas and frozen ET cycles

Characteristics	Outcomes
Age (year)	35.8 ± 3.8
Infertility factor (%)	
Male factor	26.2
Tubal factor	29.2
Ovarian factor	22.9
Multiple factors	21.7
No. of FET cycles	180
No. of FET cycles include only thawed morulas ^a^	128
Insemination methods (%)	
Conventional insemination	67.0
Intracytoplasmic sperm injection	33.0
Endometrial preparation methods (%)	
Natural cycle	17.8
Artificial hormone replacement	82.2
No. of surplus morulas thawed	347
Morula development stage at fresh cycle	
No. of day 5 morulas (%)	329 (94.8%)
No. of day 6 morulas (%)	18 (5.2%)
Morula grading at fresh cycle	
No. of M1 (%)	54 (15.5%)
No. of M2 (%)	138 (39.8%)
No. of M3 (%)	155 (44.7%)
Morula post-thaw survival rate (%)	79.3
Morula to blastocyst formation rate (%)	66.6
Morula to top-quality blastocyst formation rate (%)	23.6
No. of morulas thawed per FET cycle	2.1 ± 0.7
No. of embryos transferred per FET cycle	1.7 ± 0.6
Clinical pregnancy rate (%)	43/128 = 33.6%
Implantation rate per transferred embryos (%)	51/220 = 23.2%
Ongoing pregnancy rate (%)	35/128 = 27.3%
Live birth rate (%)	29/128 = 22.7%
Abortion rate (%)	7/43 = 16.3%
Multiple pregnancy rate ^b^ (%)	7/43 = 16.3%

^a^Excluding cycles in which embryos transferred were from mixed frozen-thawed blastocysts and cancelled cycles

^b^Including two sets of monozygotic twins

The day 5 morulas graded as M1, M2, and M3 had BFRs of 88.9, 74.0, and 52.8% (*p* < 0.001), respectively, and they had top-quality blastocyst formation rates of 64.8, 25.2, and 9.0% (*p* < 0.001), respectively. None of the day 6 morulas were graded as M1. The top-quality BFR for the day 6 morulas was 5.6% (1/18) (Table 2).

**Table 2:** Day 5 and day 6 morulas and their ability for blastocyst development in the following 1 day of in vitro cultivation after freezing-thawing procedure

	Day 5 morulas (*n* = 329)				Day 6 morulas (*n* = 18)			
	M1	M2	M3	*P*	M1	M2	M3	*P*
Morula No.	54	131	144		0	7	11	
BFR^a^, %	48/54, 88.9%	97/131, 74.0%	76/144, 52.8%	<0.001		3/7, 42.9%	3/11, 63.6%	NS
Top^b^ BFR, %	35/54, 64.8%	33/131, 25.2%	13/144, 9.0%	<0.001		0/7 0%	1/11 9.1%	NS

^a^*BFR* Blastocyst formation rate. ^b^ Top, >3AA blastocysts. *NS* Not significant

Regression analysis was employed to determine whether different variables could predict which slow-growing morulas develop into blastocysts. The variables included in the analysis were age, insemination methods, whether the morula was at day 5 or day 6, and morula grading before vitrification. Morula grading was the only variable that was found to predict blastocyst formation (M2, odds ratio 0.305; 95% confidence interval, 0.111–0.843. M3, odds ratio 0.114; 95% confidence interval, 0.042–0.311) (Table 3).

**Table 3:** Regression analysis/prediction of the development of morulas to blastocysts

Variable	Significance	OR	95% CI for OR	
			Lower	Upper
Morula grades				
M1	–	1	–	–
M2	0.022	0.305	0.111	0.843
M3	<0.001	0.114	0.042	0.311
Age	0.137	0.950	0.888	1.016
Insemination method				
Conventional IVF	–	1	–	–
ICSI	0.487	0.828	0.487	1.409
Compaction day				
Day 5	–	1	–	–
Day 6	0.492	0.701	0.255	1.930

*OR* Odds ratio, *CI* Confidence interval, *ICSI* Intracytoplasmic sperm injection

### Pregnancy outcomes

When analyzing pregnancy outcomes, the 43 FET cycles in which thawed morulas were mixed with thawed blastocysts were excluded, as were the nine cancelled cycles. Pregnancy outcomes were therefore analyzed in the 128 FET cycles that included only morulas that underwent the freezing-thawing process. The clinical pregnancy rate per FET cycle was 33.6%. The abortion rate was 16.3%. The rate of multiple pregnancies was 16.3%. Other pregnancy outcomes are summarized in Table 1. Of the 29 pregnancies that resulted in live births, six were from thawed day 5 M3 morulas. None of the embryos derived from thawed day 6 morulas resulted in a positive pregnancy outcome. The average numbers of blastocysts and top-quality blastocysts contained in transferred embryos were larger for the positive clinical pregnancy cycles (*n* = 43) than for the negative cycles (*n* = 85). There was no significant difference in cycles containing embryos from top-quality morulas (M1) and the numbers of total embryos transferred (Table 4).

**Table 4:** Characteristics of cycles with positive and negative clinical pregnancy

Variable	Positive pregnancy, *n* = 43	Negative pregnancy, *n* = 85	*P*
Age, year	35.8 ± 3.2	35.9 ± 3.6	NS
Endometrial preparation, hormone replacement, %	74.4	82.4	NS
Peak endometrium thickness, cm	1.1 ± 0.3	1.1 ± 0.2	NS
Containing embryos from top morula (M1), n (%)	16 (37.2)	26 (30.6)	NS
No. of embryos transferred per cycle, n	1.8 ± 0.6	1.7 ± 0.5	NS
No. of blastocysts^a^ contained in transferred embryos	1.7 ± 0.7	1.4 ± 0.6	0.023
No. of top blastocysts^a^ contained in transferred embryos	0.9 ± 0.7	0.4 ± 0.6	<0.001

^a^Blastocysts developed from thawed morulas

*NS* Not significant

## Discussion

The study demonstrated the feasibility of freezing poor-quality surplus morulas on day 5/day 6. Following thawing of the morulas and an additional day of culture, satisfactory survival and blastocyst formation rates were observed.

To our knowledge, the developmental potential of delayed or incompletely compacted morulas in frozen-thawed cycles has never been systematically described. This pilot study revealed results obtained from surplus morulas, which showed a BFR as high as 66.6%; this rate is comparable to the BFR of normally developed day 4 morulas during fresh cycles (68.5%) [3]. Our study found that over one-half of heavily fragmented morulas (M3) could develop into blastocysts, which showed a higher BFR than what has been previously reported. In 2011, Ivec M et al. found the BFR of day 5 morulas to be 84% in the fresh cycle [9]. In poor-quality day 5 morulas (> 20% fragments), the optimal (5AA at least) blastocyst formation rate was 13.6% [9]. Our surplus poor-quality day 5 morulas (> 10% fragments) had a 16.7% top-quality (3AA at least) blastocyst formation rate after the thawing procedure. The study details from previous publications discussing different conditions in morulas are summarized along with our pilot study results (See Additional file 1: Table S1).

**Fig. 1:**
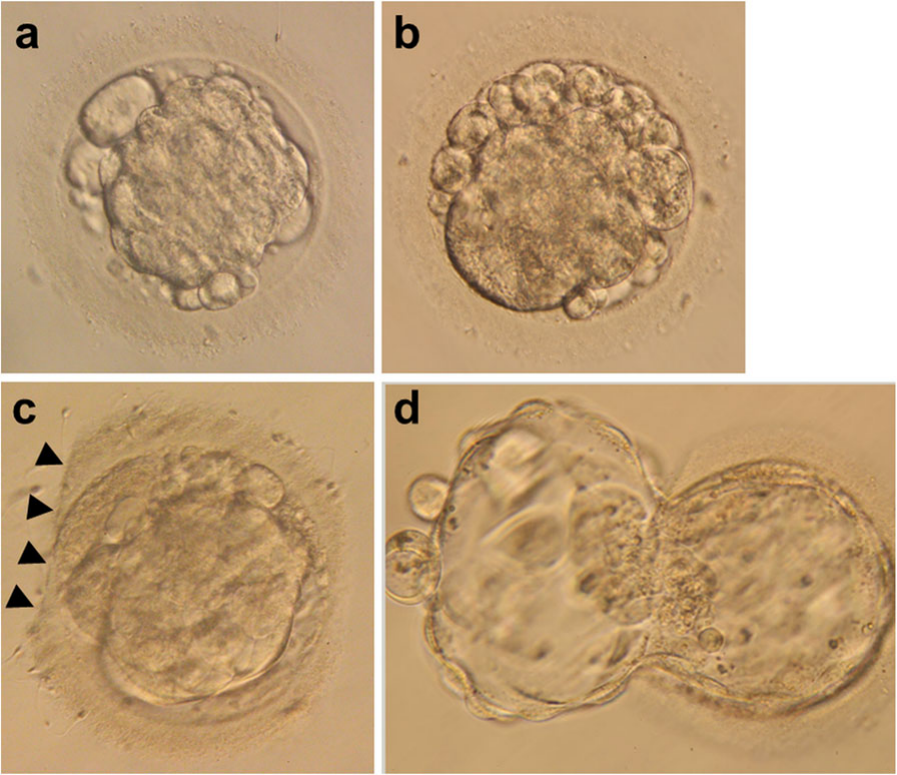
M3 morulas in different periods. **a** M3 morula in the fresh cycle just before vitrification. **b** M3 morula in the FET cycle immediately after thawing. **c** Thawed M3 morula after laser-assisted hatching; the straight dissection on the zona pellucida (arrowheads) is present. **d** 5AA blastocyst evolved from thawed M3 morula

Since the freezing-thawing strategy was modified, we focused on the development of heavily fragmented morulas following this procedure. The degree of embryo fragmentation is closely related to chromosome anomalies [14]. Fragmentation of cleavage stage embryos is an indicator of poor blastocyst development [11]. The presence of significant numbers of fragments, particularly in conjunction with discrepancies in blastomere symmetry, substantially reduces embryo viability and has a negative impact on clinical outcomes. Studies have suggested that if cellular fragmentation leads to apoptosis or limits the rate of blastomere cleavage, the removal of these fragments might improve cell division and implantation [13, 26]. The selection of human embryos that survive cryopreservation and continue to undergo cleavage in vitro has been shown to significantly improve the delivery rate [27]. It can be hypothesized that anucleate cell fragments that exhibit erratic distribution of the cell adhesion protein E-cadherin cannot survive the freezing-thawing procedures [13]. Moreover, laser-assisted hatching may benefit morulas by expelling fragmented debris and may improve cell division and blastocyst formation [7, 28]. We discovered that the fragments can be removed/released more easily with laser-assisted hatching after thawing (Fig. 1). Further study should explore whether this phenomenon can influence outcomes.

It may be an option to let the delayed morulas declare their growth capacity in fresh cycles, with increasing literature having demonstrated that day 6/7 blastocysts achieved live births [29–32]. However, concerning the earlier implantation window in the stimulated cycle, the slow-developing embryos often miss the synchronized endometrium and are cryopreserved [4, 5, 29]. Morula stage embryos have undergone genome activation [3]; referring to the adequate post-thaw BFRs, our policy of cryopreserving surplus morulas enabled the laboratory process and offered a flexible schedule for the following FET for both the couple and the clinicians. The manipulations and medical records of a couple can be completed on the same day in most circumstances, which reduces mistakes. The morulas used in these studies were surplus, which indicated that most of the couples had other embryos frozen. The couples did not pay extra fees for vitrification and storage at our institution. Further randomized controlled studies may be required to compare the BFRs of day 5/6 morulas in fresh and FET cycles and to explore the pregnancy outcomes following extended culture of surplus morulas after undergoing the freezing-thawing procedure (See Additional file 2: Table S2).

Unlike the day 3 cleavage and day 5 blastocyst stages, no consensus has been reached about the embryo morphological scoring criteria for morulas [1, 3, 8, 9, 15, 21, 28, 33]. This may have contributed to the neglect of morulas in research. A consensus has been reached about morphologic criteria at the blastocyst stage, with the transfer of good-quality blastocysts resulting in better implantation potential and better clinical outcomes than what is achieved with other blastocysts [34–36]. A sophisticated scoring system for morulas had a better predictive value than the traditional scoring system based on the day 3 cleavage stage [3]. The grading system proposed in the present study was based on compaction and fragmentation percentages. The BFR and top-quality BFR were significantly different and had a decreasing trend among M1 to M3 in day 5 morulas (Table 2). The predictive value for blastocyst formation was good, and the scoring principle was easy to remember (Table 3).

A higher aneuploidy rate has been reported in slow-growing embryos [37]. Expanded blastocysts at day 5 had a higher chance of live birth rate than those at day 6 after thawed blastocyst transfer [38]. The embryo expansion day as well as the morphology need to be given full consideration when selecting embryos. During the study period, 18 day 6 morulas were thawed. Only one top-quality blastocyst was formed from these morulas, in which no positive pregnancy outcomes were reported. The aneuploidy rate of day 6 compacted morulas obtained during fresh cycles from women aged > 38 years was 97% [31]. Further studies into the cost-effectiveness of biopsy and/or the vitrification of day 6 morulas are required, and the decision-making process should be shared with the patients.

In our study, we analyzed the post-thaw developmental potential of slow-growing poor-quality morulas. We graded the morulas and found a linear trend of the BFR among M1, M2 and M3. However, this study had several limitations. First, embryo viability was assessed by morphology alone. Additional methods, such as morphokinetics and metabolism, may be incorporated into this strategy. Second, the embryo assessment is subjected to interoperator variability, and until now, there have been no objective methods or commercial tools available for measuring embryo fragments. Third, preimplantation genetic screening (PGS) was not applied to our cohort; therefore, the euploidy rate of these surplus morulas was unclear. Fourth, the cost-effectiveness of this strategy was difficult to define, so further randomized controlled trials may be required (see Additional file 2: Table S2).

This proof-of-concept study indicates that the described strategy should not be overlooked and may be applicable to the schedule of high-volume IVF laboratories (i.e., the surplus embryos can be cryopreserved on the same day with fresh embryo transfer and weekend work can be avoided). In clinical practice, programming is very convenient, both for physicians by facilitating their work schedules and for patients by enabling them to plan appointments. We suggest that the morulas be cryopreserved in separate containers from blastocysts to facilitate single embryo transfer (SET) or further cultivation. Regarding the high top-quality BFR of day 5 M1 morulas, we suggest cryopreserving them individually, which will prevent revitrification. In this study, we did not follow through with SET because the average age of the women was 35.8 years (> 35 years), there was a lack of PGS, and most of the outcomes from the fresh cycles proved to be a failure.

## Conclusions

The post-thaw blastocyst formation rate was satisfactory, with approximately one-half of heavily fragmented morulas (M3) developing into blastocysts. Most of the poor-quality morulas were worth to freeze, with the reasonable goal of obtaining pregnancy and live birth. This alternative strategy may be a feasible approach for coping with poor-quality surplus morulas in non-PGS cycles.

## Supplementary information

**Additional file 1: Table S1.** Summary of literature review and our study about morula stage.

**Additional file 2: Table S2**. Proposals of future randomized controlled studies.

## Data Availability

The datasets used and/or analyzed during the current study are available from the corresponding author on reasonable request.

## Abbreviations

BFR: Blastocyst formation rate
FET: Frozen embryo transfer
hCG: Human chorionic gonadotropin
ICSI: Intracytoplasmic sperm injection
IVF: In vitro fertilization
PGS: Preimplantation genetic screening
SET: Single embryo transfer
