# Supplementary material for: Developmental potential of surplus morulas with delayed and/or incomplete compaction after freezing-thawing procedures
Source: Reprod Biol Endocrinol. 2019 Oct 30;17:87. doi: 10.1186/s12958-019-0535-2 (PMC6821030; doi:10.1186/s12958-019-0535-2)
Supplement: Supplementary file 1 — Additional file 1: Table S1. Summary of literature review and our study about morula stage. [file 12958_2019_535_MOESM1_ESM.docx]

**Additional file 1: Table S1.** Summary of literature review and our study about morula stage.

| Authors and publication year | Studied morulas and stages | Main outcome measurement | Strengths | Limitations |
| --- | --- | --- | --- | --- |
| Fabozzi *et al.*, 2016 [3] | Day 4 morulas in fresh cycles | - BFR/top-quality BFR per morula category | Better BFR prediction at the morula stage with respect to day 3. | - No data on FET cycles - No data on delayed morulas |
| Ivec *et al.*, 2011 [9] | Day 5 morulas in fresh cycles | - BFR/optimal BFR per morula grade | Analyzed the growth potential of delayed and/or incomplete compaction morulas. | - No data on FET cycles - No differences of BFR among day 5 grade I/II/III morulas |
| Haas *et al.*,  2019 [4] | Day 5 morulas in fresh cycles | - BFR (blastulation rate) | Large number of embryos. | - No morulas grading - No data on FET cycles |
| Tao *et al.*, 2004 [10] | Day 4 morulas in  FET cycles | - Post-thaw morula survival rate - Pregnancy rates | Reported the survival rate of post-thaw morulas, its morphological alterations during freezing-thawing procedures and outcomes after FET. | - No data for delayed morulas - No information about BFR of the morulas |
| Tsai *et al.,*  2019 | Day 5/6 morulas in FET cycles | - Post-thaw morula survival rate - BFR/top-quality BFR per morula grade - Pregnancy rate | Reported the growth potential of delayed and/or incomplete compaction morulas in FET cycles. | - Further studies may be required to compare the BFRs of day 5/6 morulas in fresh and in FET cycles. |

BFR, blastocyst formation rate; FET, frozen embryo transfer.
