# Supplementary material for: Developmental potential of surplus morulas with delayed and/or incomplete compaction after freezing-thawing procedures
Source: Reprod Biol Endocrinol. 2019 Oct 30;17:87. doi: 10.1186/s12958-019-0535-2 (PMC6821030; doi:10.1186/s12958-019-0535-2)
Supplement: Supplementary file 2 — Additional file 2: Table S2. Proposals of future randomized controlled studies. [file 12958_2019_535_MOESM2_ESM.docx]

**Additional file 2: Table S2.** Proposals of future randomized controlled studies.

| Design | Intervention group | Control group | Outcome analysis |
| --- | --- | --- | --- |
| Design 1 | In FET cycles, culturing day 5/6 morulas for an additional day. | In fresh cycles, culturing day 5/6 morulas for an additional day. | Blastocyst formation rate  Top blastocyst formation rate |
| Design 2 | In FET cycles, further culturing day 5/6 morulas and transferring embryos on the following day. | In FET cycles, transferring thawed day 5/6 morulas on the same day. | Implantation rate  Live birth rate  Abortion rate  Multiple pregnancy rate |

FET, frozen embryo transfer.
